# Altered Molecular Expression of the TLR4/NF-κB Signaling Pathway in Mammary Tissue of Chinese Holstein Cattle with Mastitis

**DOI:** 10.1371/journal.pone.0118458

**Published:** 2015-02-23

**Authors:** Jie Wu, Lian Li, Yu Sun, Shuai Huang, Juan Tang, Pan Yu, Genlin Wang

**Affiliations:** College of Animal Science and Technology, Nanjing Agricultural University, Nanjing, People’s Republic of China; IISER-TVM, INDIA

## Abstract

Toll-like receptor 4 (TLR4) mediated activation of the nuclear transcription factor κB (NF-κB) signaling pathway by mastitis initiates expression of genes associated with inflammation and the innate immune response. In this study, the profile of mastitis-induced differential gene expression in the mammary tissue of Chinese Holstein cattle was investigated by Gene-Chip microarray and bioinformatics. The microarray results revealed that 79 genes associated with the TLR4/NF-κB signaling pathway were differentially expressed. Of these genes, 19 were up-regulated and 29 were down-regulated in mastitis tissue compared to normal, healthy tissue. Statistical analysis of transcript and protein level expression changes indicated that 10 genes, namely TLR4, MyD88, IL-6, and IL-10, were up-regulated, while, CD14, TNF-α, MD-2, IL-β, NF-κB, and IL-12 were significantly down-regulated in mastitis tissue in comparison with normal tissue. Analyses using bioinformatics database resources, such as the Kyoto Encyclopedia of Genes and Genomes (KEGG) pathway analysis and the Gene Ontology Consortium (GO) for term enrichment analysis, suggested that these differently expressed genes implicate different regulatory pathways for immune function in the mammary gland. In conclusion, our study provides new evidence for better understanding the differential expression and mechanisms of the TLR4 /NF-κB signaling pathway in Chinese Holstein cattle with mastitis.

## Introduction

Mastitis is the persistent inflammatory response of mammary tissue attributed to intra-mammary invasion of pathogens [1]. Many different microbial and environmental factors can induce mastitis, so, it is important to understand the mechanisms controlling the immune response at the molecular level [2]. A better understanding of the molecular events and transition periods in response to different pathogens [1] would provide mechanistic insight into the physiological changes that render the mammary gland more susceptible to mastitis. This, in turn, would fuel the development of better preventative measures and bioinformatics-based approaches for data mining [3].

Toll-like receptor 4 (TLR4), a pattern recognition receptor, plays an important role in the induction of the inflammatory response by recognizing exogenous pathogen-associated molecular patterns and endogenous ligands [4, 5]. Activation of TLR4 is linked to the expression of pro-inflammatory cytokines and the activation of nuclear factor kappa B (NF-κB) signaling pathways in several cell types [6, 7]. It was reported that TLR4 expression positively correlates with the levels of tumor necrosis factor-α(TNF-α) and interleukin-6 (IL-6) in models of myocardial ischemia-reperfusion rats [8]. In dairy cattle, increased NF-κB activity was found in the milk and intra-mammary epithelial cells of mastitis-affected cows. The binding of lipopolysaccharide (LPS) to TLR4, in complex with CD14 and LY96 (Lymphocyte antigen-96; also known as MD-2), initiates TIR (Toll/IL1 Receptor) domain intracellular signaling through adaptor molecules, predominantly myeloid differentiation actor 88 (MyD88) [9]. This TLR4 and MYD88 signaling results in the activation of downstream kinases, leading to the degradation of IKB, which frees NF-κB to translocate to the nucleus. There, it binds κB sites in the promoter region of genes encoding pro-inflammatory cytokines, including IL-1B and IL-6 [10]. Gilbert et al. [11] found that bovine mammary epithelial cells (bMEC) respond differently to various pathogenic stimuli. Crude LPS from *Escherichia coli* was associated with an NF-κB and Fas signaling pathway, while the response to *Staphylococcus aureus* culture supernatant (SaS) was associated with an AP-1 and IL-17A signaling pathway. Sipka et al. [12] investigated the effect of intra-mammary treatment with cephapirin, alone or in combination with prednisolone, on gene expression profiles in mastitis experimentally-induced by *E.coli* in Holstein Friesian cows. They found that both treatments resulted in down-regulation of gene transcripts involved in chemokine and TLR-signaling pathways compared to challenged, untreated quarters. TLR4, which detects LPS from the cell wall of gram-negative bacteria and initiates the MyD88-IKK-NF-κB pathway response, is well established as an important cell surface receptor for the inflammatory response [13, 14]. TLR4 regulation of LPS activates the MyD88-dependent pathway (mediated by TLR–IL-1 receptor domain containing adapter protein/TIRAP), leading to the rapid activation of NF-κB, which induces the production of various pro-inflammatory cytokines [15]. In addition, Zheng et al. [16] found that thymol could reduce the internalization of *Staphylococcus aureus* into bMEC by inhibiting NF-κB activation and down-regulating the mRNA expression of tracheal antimicrobial peptide (TAP) and β-defensin (BNBD5). In the present study, we compared the gene expression of several key molecules involved in TLR4 activation and the NF-κB signaling pathway in mammary tissue from Chinese Holstein cattle with and without mastitis. The results presented help facilitate deeper insight into the specific regulatory mechanisms of bovine mastitis.

## Material and Methods

### 1. Tissue Samples

RNA samples were isolated from the mammary tissue of adult Chinese Holstein cows during late lactation with (n = 3) and without (n = 3) mastitis. The cows were received total mixed ration (TMR) premix. The Chinese Holstein cows without mastitis were not use of antibiotics, while the cows with mastitis were use. All of the cows were not injected with recombinant bovine somatotropin (rBST). Pathological changes indicating clinical mastitis were signs of udder inflammation, including dolor, rubor, change of color, and visibly abnormal milk containing flakes. The presence or absence of clinical mastitis was established by somatic cell count (SCC), the California mastitis test (CMT), and hematoxylin-eosin staining of mammary gland tissue (S1 Appendix). The experimental protocol used in this study was approved by the Ethics Committee of NanJing Agricultural University, China, and was performed in accordance with the Animal Care and Use Statute of China. Mammary tissue was collected within 10–20 min after slaughter, and was frozen in liquid nitrogen until RNA extraction. Tissue was carefully segregated using ophthalmology tweezers and scalpels to avoid contamination.

### 2. RNA isolation

Total RNA was extracted using Trizol reagent (Invitrogen, Carlsbad, USA) according to the manufacturer’s protocol. The absorbance values at 260 and 280 nm were obtained to assess RNA concentration and purity in the samples. RNA integrity was assessed by electrophoresis on 2% agarose gels (m/v).

### 3. Microarray assay

Gene chip analysis of the Bovine Genome Array was performed by an outside service provider (LC-Bio. CO., LTD). Total RNA from the tissue specimens was individually hybridized with gene chips. Briefly, in the first-strand cDNA synthesis reaction, 10 mg total RNA was used for reverse transcription using a T7-oligo (dT) promoter primer. Then, the double–stranded cDNA was synthesized from the first-strand cDNA using RNase H. After purification of the resulting DNA, an in vitro transcription reaction using the MEGA Script T7 Kit (Ambion, Inc., USA) produced biotin-labeled cRNA. After the cRNA was cleaned and fragmented, it was hybridized to the probe array at 45°C for 16 h. Thereafter, the probe array was washed and stained on the Fluidics Station, and the microarrays were scanned using a GeneChip Scanner 3000 (Affymetrix). The Affymetrix Micro Array Suite 5.0-Specific Terms GCOS version 1.4 was used for quantity analysis of the hybridization. The gene expression levels that had ≥2-fold difference between normal and mastitis tissue were checked and further analyzed. The Molecule Annotation System (http://david.abcc.ncifcrf.gov/) was used to analyze the differentially expressed genes using the Kyoto encyclopedia of genes and genomes (KEGG) public pathway resource and the Gene Ontology (GO) consortium. The microarray data has been deposited in the Gene Expression Omnibus database (accession number: GSE62845).

### 4. Real-time reverse transcription-polymerase chain reaction (RT-PCR)

RT-PCR was performed to confirm the microarray results. Total RNA was extracted from normal and mastitis mammary tissue, as described above, and total RNA was reverse transcribed using a Reverse Transcription Levels kit (TaKaRa, Dalian, China) according to the manufacturer’s protocol. The expression levels of 10 genes were measured. The house keeping gene 18S rRNA was used as an endogenous control.18s rRNA is stable in all cases and low regulation by external influence, so the background is more stable when in RT-PCR. Studies have shown that 18s rRNA has previously been used as reference genes for qPCR with stimulated MEC [17, 18]. The 18s rRNA gene has previously been found suitable as reference genes for bovine cells and tissues [19]. Primers were designed using Primer Premier 5.0 and are shown in Table 1. RT-PCR was performed with SYBR Premix Ex Taq (TaKaRa Biotechnology Co., Ltd., Japan). The reaction solution was prepared on ice, and consisted of: 10 μL of 2×SYBR Premix Ex Taq, 0.8μL of PCR Forward Primer (10 μM), 0.8μL of PCR Reverse Primer (10 μM), 0.4 mL of 50×ROX Reference Dye, 2 μL of cDNA (100 ng μL^-1^), and dH2O to a final volume of 20 μL. The reaction mixtures were incubated in a 96-well plate at 95°C for 30 s, followed by 40 cycles of 95°C for 5 s, 60°C for 30 s and 72°C for 30 s. All reactions were performed in triplicate. The gene expression levels were analyzed with the 2^-ΔΔCT^ method.

**Table 1:** Primer sequences for RT-PCR.

Gene name	NCBI Accession number	Product size (bp)	Primer Sequence (5’-3’) Sense/antisense
IL-6	NM_173923.2	153	TGCTGGTCTTCTGGAGTATC
			GTGGCTGGAGTGGTTATTG
IL-1β	NM_174093.1	264	CGTCTTCCTGGGACATTTTCG
			GTCTGAGGATGGGCTCTGGG
MD-2	AB072456.1	276	ACCGTTTGGTACGACTACTGTGAT
			CCTGAAGGAGAATTGTATTGTTGTG
NF-kB	XM_005226181.1	249	AAGAGAAGATGGGGAAAGGCTG
			CGTCGGCAAATGAGAAGTAGTG
IL-12	AJ308426.2	238	ATTTATGTTCAGCATGGTCACTCC
			CAGTGTACAGGTGGGTGTCTCG
LBP	NM_001038674.2	108	TCAAGGGCATCACCAT
			GCACCTCCACATCACG
CD14	NM_174008.1	109	ACCACCCTCAGTCTCCGTAA
			GTGCTTGGGCAATGTTCAG
MyD88	NM_001014382.2	177	GCAGCATAACTCGGATAAA
			CAGACACGCACAACTTCA
TLR4	NW_003103900.1	166	TCCCACATCCTCGGTTCCC
			TCCATCCCAAGCCATCCCT
TNF-a	NM_173966	175	ACCCAGCCAACAGAAGC
			CCAGACGGGAGACAGGA
18S rRNA	NM_174841.2	116	TCCAGCCTTCCTTCCTGGGCAT
			GGACAGCACCGTGTTGGCGTAGA
IL-10	NM_174088	110	GCCACAGGCTGAGAACC
			TTTCACTGCCTCCTCCAGAT

### 5. Protein isolation and Western blotting

Total protein was extracted from normal and mastitis tissue using the Nuclear and Cytoplasmic Protein Extraction Kit (thermo) according to the manufacturer’s instructions. Protein was transferred onto polyvinylidene fluoride membranes and blocked overnight in 5% BSA in Tris-buffered saline with Tween 20 at 4°C. Membranes were incubated for 2 h at room temperature with the appropriate primary antibody. After three 15-min washes in Tris-buffered saline with Tween20, membranes were incubated with an appropriate secondary antibody conjugated to horseradish peroxidise diluted 1:1000 in block solution for 2 h. After three washes, blots were developed with ECL substrate using the Western Blotting Detection System (Amersham Life Science,UK).

### 6. Statistical analysis

All data were obtained from one independent experiment carried out in triplicate. The fold changes of genes between healthy and mastitis cows were calculated using fold Change = 2^−ΔΔCt(mastitis-healthy)^ = 2 ^−(ΔCt(mastitis)−ΔCt(healthy))^ [20]. Data are presented as mean + SEM, main and interactive effects were analyzed by the independent-samples t-test using SPSS16.0 software. P< 0.05 was considered statistically significant.

## Results

### 1. Differentially expressed genes

**Fig 1:**
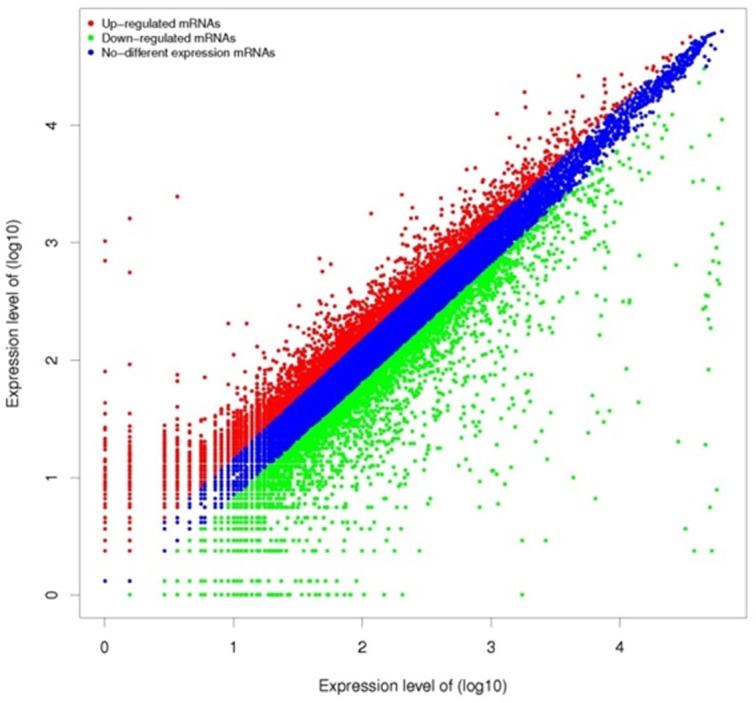
Log-Log Scatter Plot of normal and mastitis mammary tissues. Red replaces higher expressed genes in mastitis tissue. Green replaces the higher expressed genes in normal tissue.

**Fig 2:**
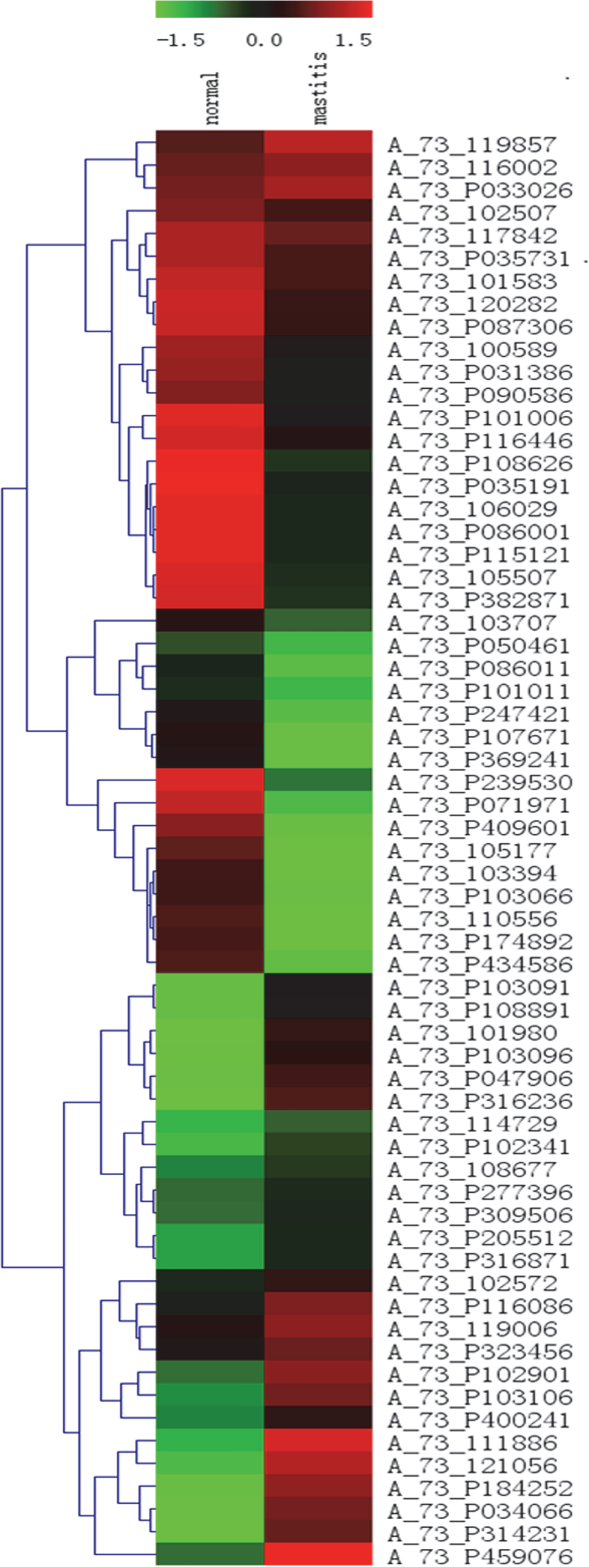
Analysis of 79 differently expressed genes of TLR4/ NF-kB signaling pathway in normal and mastitis mammary tissues (*P*<0.01). The microarray heat map demonstrates the log2 ratio of gene signal difference between normal and mastitis tissues. Up-regulated genes are shown in red, while down-regulated genes are shown in green. The left column on the figure represents the level of expression in normal mammary tissue, while the right column represents the level of corresponding gene expression in mastitis mammary tissue.

After quantifying all hybridization spots, the signal intensity was plotted logarithmically. Fig. 1 shows the scatter plot of microarray signals from normal and mastitis tissue. The expression levels of many genes differed between groups. Microarray data analysis indicated that 79 genes associated with the TLR4/NF-kB signaling pathway were differentially expressed (P < 0.01) (Fig. 2). Comparison of the two groups revealed that 19 genes from the TLR4/NF-kB signaling pathway were up-regulated (≥2 fold), whereas, 29 genes were down-regulated in mastitis tissue (≥2 fold) (Table 2).

**Table 2:** Differentially expressed genes in mastitis mammary tissues with respect to normal mammary tissues.

Gene symbol	Representative public ID	Normal	Mastitis	Fold-change [log2(m/n)]
Up-regulated genes in mastitis mammary tissues				
TICAM2	NM_001046456	8.05	9.35	1.30
PIK3CA	NM_174574	4.63	5.96	1.33
STAT1	NM_001077900	0.65	2.06	1.41
IL10	NM_174088	10.06	11.55	1.49
IKBKG	NM_174354	-6.64	-4.98	1.66
MAPK11	NM_001080335	-6.64	-4.98	1.66
IFNAR2	NM_174553	2.48	6.85	1.77
TLR6	NM_001001159	2.48	3.32	1.78
CASP8	NM_001045970	-1.41	0.40	1.81
TAB2	NM_001192372	-1.41	0.40	1.81
MAPK8	NM_001192974	1.65	4.29	2.75
MAP2K2	NM_001038071	-1.41	2.06	3.47
MAPK10	NM_001083728	-1.41	2.48	3.90
MyD88	NM_001014382	-4.98	1.25	6.23
TLR9	NM_183081	-6.64	0.40	7.04
IRAK1	NM_001040555	-4.98	2.58	7.56
TLR4	NM_174198	-6.64	2.19	8.83
TLR7	NM_001033761	-6.64	2.58	9.22
IL6ST	XM_600430	-1.41	0.40	1.81
Down-regulated genes in mastitis mammary tissues				
CD14 molecule	NM_174008	2.48	-4.98	-7.47
CXCL11	CK959898	1.87	-4.98	-6.86
MAPK14	NM_001102174	1.87	-4.98	-6.86
TLR3	NM_001008664	0.65	-4.98	-5.63
MAP2K3	NM_001083693	9.31	3.84	-5.47
NFKB1	NM_001076409	11.56	7.84	-3.72
IFNAC	NM_174085	-1.41	-4.98	-3.57
IFNW1	NM_174351	-1.41	-4.98	-3.57
MAP2K6	NM_001034045	-1.41	-4.98	-3.57
TLR8	NM_001033937	-1.41	-4.98	-3.57
CXCL10	NM_001046551	6.49	2.98	-3.51
IRF5	NM_001035465	3.79	0.40	-3.40
TNF	NM_173966	7.72	5.00	-2.72
IL12A	CB168830	5.96	3.54	-2.42
AKT1	NM_173986	3.32	1.25	-2.07
RELA	NM_001080242	5.21	3.32	-1.89
IRF7	NM_001105040	11.28	9.46	-1.82
IKBKE	NM_001046345	2.19	0.40	-1.79
NFKBIA	NM_001045868	5.40	3.66	-1.74
TLR1	NM_001046504	4.78	2.58	-1.65
JUN	NM_001077827	5.40	4.25	-1.54
TBK1	NM_001192755	5.79	3.32	-1.45
MAPK13	NM_001014947	5.96	4.82	-1.38
IL1B	NM_174093	7.43	6.14	-1.29
MAP3K7	NM_001081595	5.79	4.51	-1.29
PIK3CD	NM_001205548	12.65	11.45	-1.19
FOS	NM_182786	1.54	0.40	-1.15
IRAK4	NM_001075998	3.19	2.06	-1.13
IRF3	NM_001029845	3.62	2.58	-1.04

### 2. Results of GO and KEGG analyses

In order to clarify the different biological patterns of the two groups, genes of the TLR4/NF-kB signaling pathway were individually analyzed by GO and KEGG, with criteria for significance set at P< 0.01.

**Fig 3:**
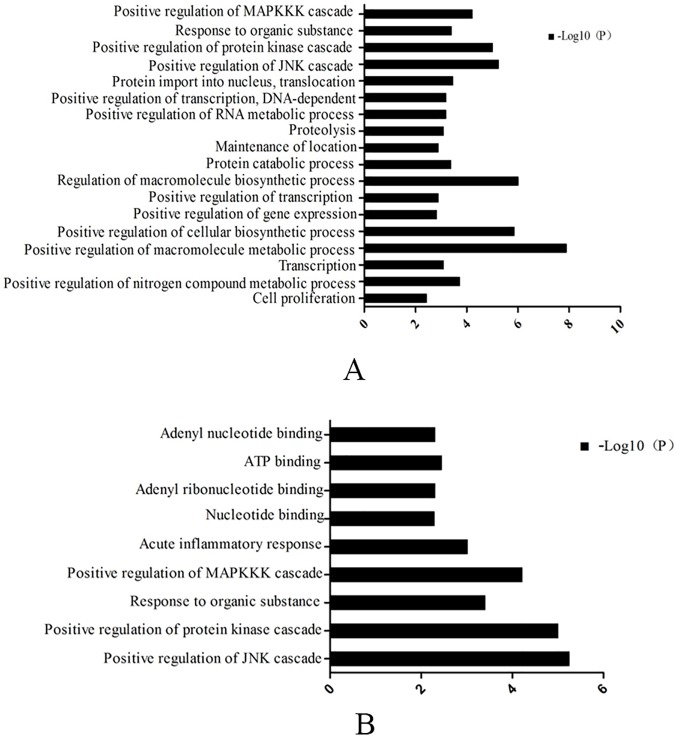
Biologial process analyzed of genes that changed two-fold ≥ 2 folds in normal (A) and mastitis mammary tissue (B) by Gene ontology analysis (P values < 0.01).

GO analysis (Fig. 3A) showed that the up-regulated genes in the mammary tissue from healthy cows were largely associated with: MAPKKK cascade, protein kinase cascade, JNK cascade, macromolecule biosynthetic process, cellular biosynthetic process, macromolecule metabolic process, etc. The up-regulated genes in the mammary tissue from cows with mastitis (Fig. 3B) were mainly implicated in: MAPKKK cascade, protein kinase cascade, JNK cascade, organic substance, inflammatory response, ATP binding, adenyl nucleotide binding and adenylribo-nucleotide binding.

**Fig 4:**
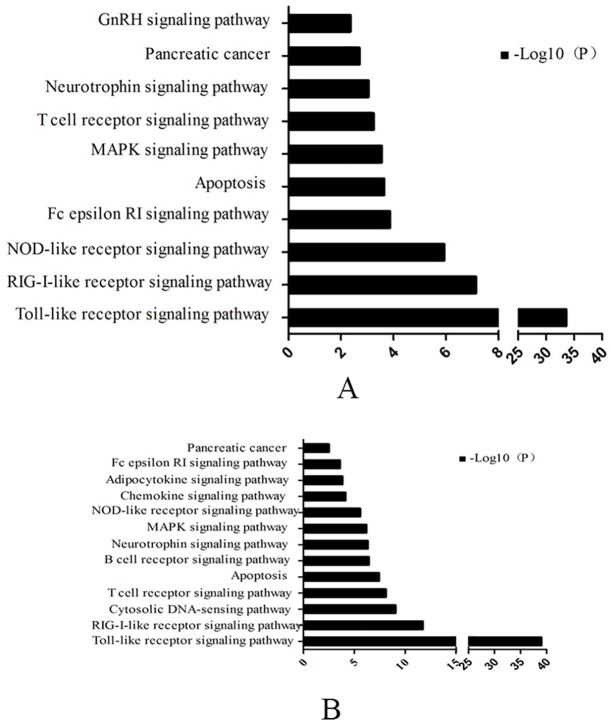
Biologial process analyzed of genes that changed two-fold ≥ 2 folds in normal (A) and mastitis mammary tissue (B) by Gene KEGG analysis (P values < 0.01).

Gene expression changes that reached statistical significance were analyzed in KEGG (Fig. 4). Fig. 4A shows that up-regulated genes in healthy mammary tissue samples are associated with the following signaling pathways: GnRH, neurotrophin, T cell receptor, MAPK, Fcepsilon RI, NOD-like receptor, RIG-I-like receptor, Toll-like receptor, pancreatic cancer and apoptosis. The up-regulated genes in tissue samples from cows with mastitis were involved in the following pathways: neurotrophin, T cell receptor, MAPK, Fc epsilon RI, NOD-like receptor, RIG-I-like receptor, adipocytokine, chemokine, B cell, cytosolic DNA-sensing Toll-like receptor signaling pathway, pancreatic cancer and apoptosis (Fig. 4B).

### 3. RT-PCR

**Fig 5:**
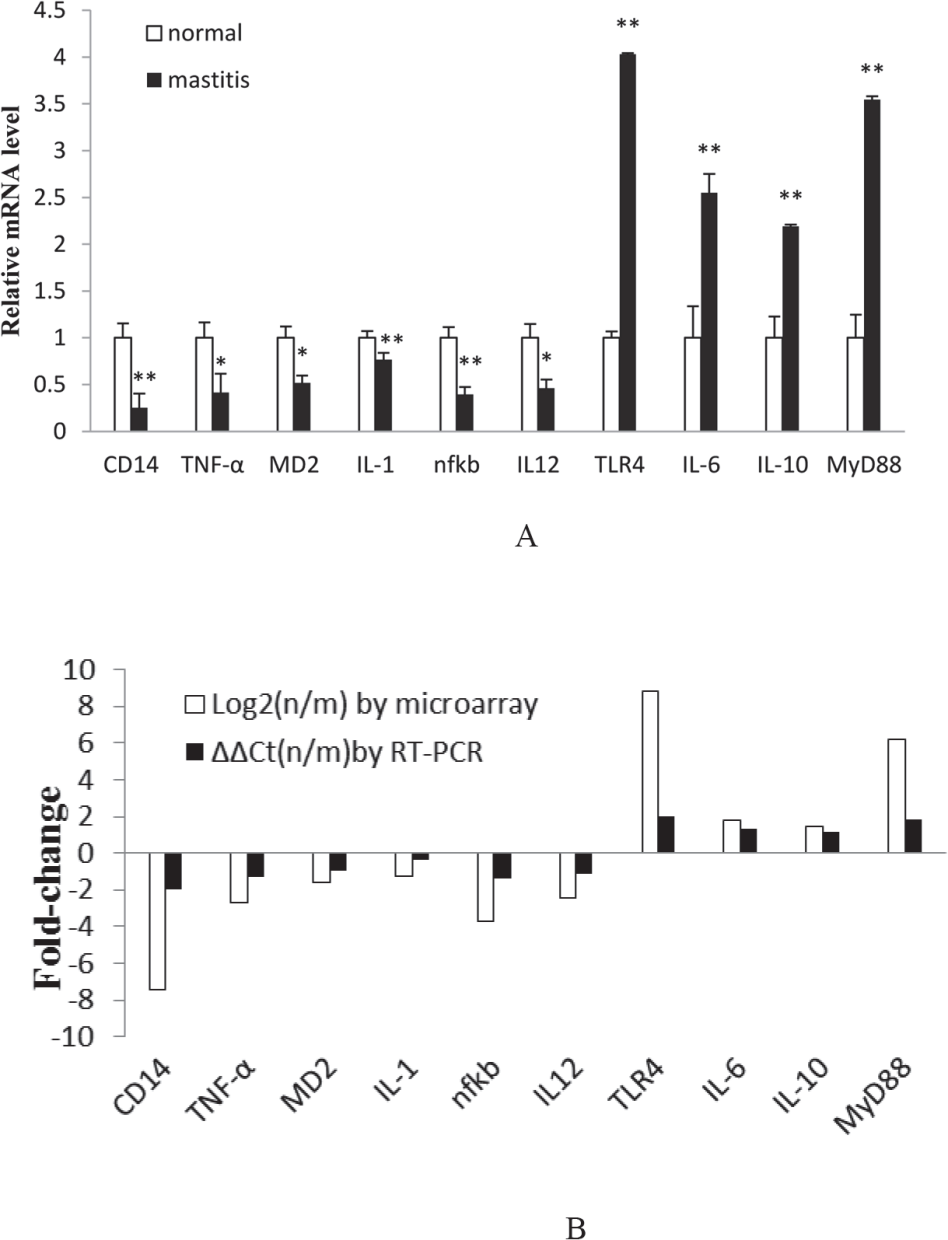
Microarray results confirmed by RT-PCR. A. RT-PCR results of genes selected. B. Comparison of RT-PCR findings to microarray results by fold-change of ten-selected genes. N = normal tissue and m = mastitis tissue. Note: a single asterisk indicates a statistical difference (*P*< 0.05), and a double asterisk indicates a statistical difference (*P*<0.01).

In order to validate the microarray chips results, 10 genes associated with the TLR4/NF-kB signaling pathway were analyzed using RT-PCR. As Fig. 5A shows, TLR4, MyD88, IL-6, and IL-10 expression levels were increased in mastitis tissue. On the contrary, CD14, TNF-α, MD-2, IL-1β, NF-kB, and IL-12 expression levels were decreased in mastitis tissue with respect to normal tissue. Thus, the RT-PCR results were in accordance with the gene chip findings (Fig. 5B).

### 4. Western blot

**Fig 6:**
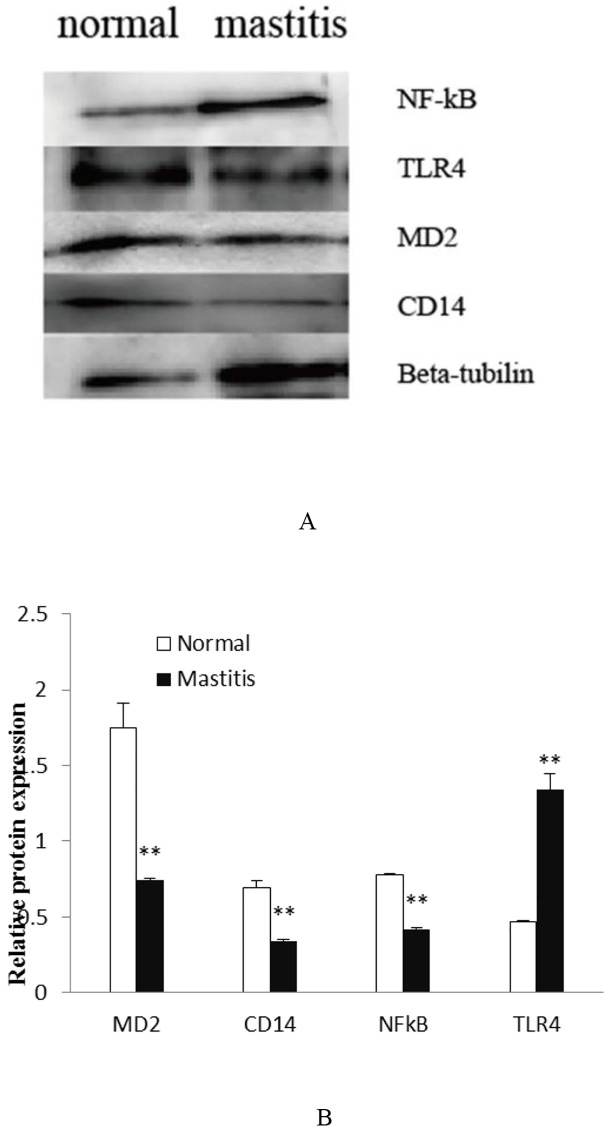
Microarray results confirmed by western blot. Note: a double asterisk indicates a statistical difference (*P*<0.01).

To examine protein level expression changes, four molecules were measured using western blot analysis. As Fig. 6 shows, CD14, MD-2, and NF-kB protein expression was decreased in mastitis tissue. While, TLR4 protein expression was increased in mastitis tissue. The Western blot results demonstrate that the protein level expression changes are consistent with changes seen at the transcript level obtained by RT-PCR.

## Discussion

Bovine mastitis, an inflammation reaction of the mammary gland, is one of the most costly diseases in the dairy industry. Mastitis can be caused by many pathogenic bacteria, such as *E. coli, Streptococcus uberis*, and *Staphylococcus aureus*. In this study, clinical mastitis was the result of infection by gram-negative bacteria. Histological assessment of mammary gland tissue from healthy cows showed that the glandular bubble chamber was full of milk and free of hyperplasia and inflammation. The mammary glands of cows with mastitis showed cavity deformation, or were filled with vacuoles of homogeneous exudate mixed with shedding epithelial and inflammatory cells. In addition, there was acini cell degeneration, atrophy, edema, and interstitial tissue hyperplasia present.

GO biological process analysis revealed that macromolecule biosynthetic, protein catabolic and cellular biosynthetic processes gather in higher levels in healthy tissue. The KEGG results revealed that genes related to the GnRH signaling pathway were more highly expressed in healthy tissue. These results imply that there are more functional associations in healthy tissue compared with mastitis tissue.

Mastitis is a serious infective disease that causes vast economic losses in the dairy industry. Alain’s survey shows that about 40–50% of clinical mastitis is caused by E.*coli* [21]. TLR4, one of the best characterized TLRs, recognizes LPS from gram-negative bacteria [22]. Activation of TLR4 is known to result in the activation of the NF-κB signaling pathway, and finally, results in the release of pro-inflammatory cytokines [23]. NF-κB is an important transcription factor located downstream of the TLR4-mediated signaling pathway. NF-κB plays an essential role in regulating the immune response, including the gene expression of many inflammatory cytokines [24]. CD14, which is found on neutrophils and macrophages in the mammary gland, is another important pathogen receptor [25]. CD14 binds LPS-protein complexes and induces the synthesis and release of TNF-α [26]. In turn, TNF-α controls the activation of two different downstream signaling pathways, AP1 and NF-κB [27]. Hence, a deficiency in TNF-α induction may impair NF-κB activation, which could subsequently turn off an important arm of the immune defense [28]. TNF-α is the earliest primary endogenous mediator of the inflammatory process [29]. IL-1β, a subtype of IL-1, can increase TNF-α levels [30]. IL-6 is a pleiotropic cytokine produced by monocytes and macrophages that is involved in vascular inflammation [31]. Fu et al. [32] found that LPS-induced mastitis in mice results in the release of pro-inflammatory cytokines such as TNF-α, IL-1β and IL-6, which can damage mammary tissue [33,34]. IL-12 is naturally produced by dendritic cells and macrophages. It has anti-angiogenic activity, which it elicits by increasing production of IFN-γ [35]. IL-12 was reported to be significantly elevated in the mammary gland 24 hours after being experimentally infected with Staphylococcus aureus in cattle [36]. In the present study, CD14, TNF-α, MD-2, IL-1β, NF-κB, and IL-12 gene expression was decreased in tissue affected by mastitis compared to normal mammary tissue, which is consistent with suppression of NF-κB activity and pro-inflammatory cytokine production. The results indicate that these immune-related genes participate in the immune response via the NF-κB signaling pathway to combat bacterial invasion.

There are many adapter proteins that participate in TLR4 signal transduction, including MyD88. Structurally, MyD88 consists of a carboxy terminal MyD88 TIR (Toll/Il-IR) domain, amino-terminal death domain (DD), and a short linker sequence. MyD88 associates with TLR4 to form a homologous dimer at the TIR and DD domains. TLR4/MyD88 signaling activates downstream kinases that degrade I κB, which normally sequesters NF-κB in the cytoplasm [24]. Once freed, NF-κB translocates to the nucleus, where it binds κB sites in the promoter region of genes encoding pro-inflammatory cytokines, including IL-6 [37]. In addition, MyD88 signaling activates the mitogen-activated protein kinase (MAPK) cascade [38]. On the other hand, IL-10, a powerful anti-inflammatory cytokine, plays an important role in improving survival in animals challenged with LPS. For example, one study demonstrated that pretreatment with Paeonol (PAE), an anti-inflammatory agent, enhanced serum IL-10 levels in LPS-challenged mice [39]. As Fig. 4A demonstrates, TLR4, MyD88, IL-6, and IL-10 were highly expressed in bovine mastitis tissue. Our results are in agreement with studies showing activation of TLR4.

Taken together, these data suggest that the immune response of Chinese Holstein cattle with mastitis may occur by a MyD88-dependent channel that is mediated by TLR4/ NF-κB. However, further studies are needed to validate and expand upon the current results.

In conclusion, the present study showed differential gene expression of the TLR4/ NF-κB signaling pathway in the mammary tissue of Chinese Holstein cattle with mastitis. Gene-chip microarray indicated that 79 genes were significantly changed compared to normal mammary gland tissue. We also analyzed the KEGG and GO term enrichment of the target genes using the DAVID bioinformatics resource and the Term enrichment tool. These data suggest that the mammary gland secretes cytokines and chemokines in response to gram-negative bacteria via the TLR4/ NF-κB pathway. Further studies may help to clarify the molecular mechanism involved in the TLR4/ NF-κB signaling pathway.

## Supporting Information

S1 Appendix The mammary gland of healthy dairy cows (A): the glandular bubble chamber was full of milk and free of hyperplasia and inflammation; the mammary gland of dairy cows with mastitis (B): as arrows showed acinus cavity deformation, or filled with vacuoles of homogeneous exudate mixed with shedding of epithelial and inflammatory cells, H.E×100. The results of milk CMT and SCC number. (DOCX)
