# Supplementary material for: Altered Molecular Expression of the TLR4/NF-κB Signaling Pathway in Mammary Tissue of Chinese Holstein Cattle with Mastitis
Source: PLoS One. 2015 Feb 23;10(2):e0118458. doi: 10.1371/journal.pone.0118458 (PMC4338248; doi:10.1371/journal.pone.0118458)
Supplement: S1 Appendix — (DOCX) [file pone.0118458.s001.docx]

**Appendix I**

Tissue sample identification

SCC data were collected at the DHI center in the Jiangsu Province. Table S1 shows that milk sample density for healthy cows ranged from 0 to 10×10^4^ cells/mL, while cows with clinical mastitis had milk sample density values above 100×10^4^ cells/ mL. The results of the CMT were consistent with the SCC values. The symbol “-” represents a homogeneous milk sample with good liquidity and a yellow color. The symbols “+” and “++” represent milk that has gelled and is yellow-green in color. The histological assessment showed that the glandular bubble chamber was full of milk and free of hyperplasia and inflammation in the mammary gland of healthy cows (Fig. S1A). The mammary glands of cows with mastitis showed cavity deformation, or were filled with vacuoles of homogeneous exudate mixed with shedding of epithelial and inflammatory cells. In addition, there was acini cell degeneration, atrophy, edema, and interstitial tissue hyperplasia present (Fig. S1B).

**Table S1.** The results of milk CMT and SCC number （×10^4^cells/mL）

| **Healthy Clinical mastitis** | | | | | | |
| --- | --- | --- | --- | --- | --- | --- |
| Number | 1 | 2 | 3 | 4 | 5 | 6 |
| SCC | 9.8 | 4.6 | 4.5 | 175.4 | 100.2 | 108.6 |
| CMT | - | - | - | ++ | + | + |


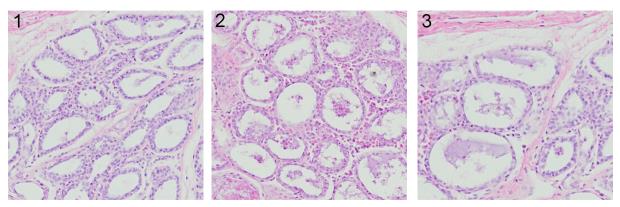

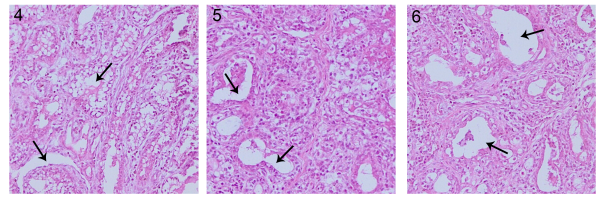


A B

**Figure S1. The mammary gland of healthy dairy cows (A):** the glandular bubble chamber was full of milk and free of hyperplasia and inflammation ; **the mammary gland of dairy cows with mastitis (B):** as arrows showed acinus cavity deformation, or filled with vacuoles of homogeneous exudate mixed with shedding of epithelial and inflammatory cells , H.E×100
